# The relationship between respiratory symptoms and frailty: findings from observational and Mendelian randomization analyses

**DOI:** 10.1007/s40520-024-02905-5

**Published:** 2024-12-30

**Authors:** Zhishen Ruan, Dan Li, Xiaodong Cong, Shasha Yuan, Yiling Fan, Bo Xu, Qing Miao

**Affiliations:** 1 Xiyuan Hospital of Chinese Academy of Chinese Medical Sciences, Beijing, China; 2 Shuguang Hospital of Shanghai University of Traditional Chinese Medicine, Shanghai, China

**Keywords:** Respiratory symptoms, Frailty index, Ageing, Cross-sectional study, Mendelian randomization

## Abstract

**Introduction:**

As ageing accelerates, frailty increasingly impacts public health. Cough, sputum, wheezing and dyspnea are common respiratory symptoms, and the relationship to frailty is unclear. We aimed to analyze the relationship between respiratory symptoms and frailty.

**Methods:**

Cross-sectional and Mendelian randomization (MR) studies were used. Cross-sectional data involved 14,021 participants from the National Health and Nutrition Examination Survey (NHANES). Logistic and linear regression were used to analyze the relationship between respiratory symptoms (cough, sputum, wheezing, dyspnea) and frailty. We adjusted for multiple variables and used propensity score matching (PSM). Mediation analysis was used to explore the role of inflammatory markers and age in the relationship between the two. We analyzed the relationship using a two-sample MR approach with data from genome-wide association studies (GWAS) to enhance causal inference.

**Results:**

Observational studies have shown that cough (OR 1.74, 95 CI% 1.44, 2.09), sputum (OR 1.87, 95 CI% 1.57, 2.22), wheezing (OR 2.01, 95 CI% 1.68, 2.40), and dyspnea (OR 2.60, 95 CI% 2.28, 2.97) are associated with an elevated risk of frailty. The PSM results were stable. Mediation analyses indicated that elevated inflammatory markers and advancing age were mediators between respiratory symptoms and frailty. The results of the MR study showed that sputum and wheezing were associated with an elevated frailty index; and in the study of FI on respiratory symptoms, all respiratory symptoms were elevated with elevated FI.

**Conclusions:**

Our study identified a potential association between frailty and respiratory symptoms. Inflammation and ageing may be essential factors mediating this association.

**Supplementary Information:**

The online version contains supplementary material available at 10.1007/s40520-024-02905-5.

## Introduction

The impact of frailty on clinical practice and public health is increasingly significant with the accelerated progress of ageing [1]. The concept of frailty originated in geriatrics and was recognized as a state of vulnerability to stress and a poor ability to maintain internal balance [2]. Weakness is a negative effect of ageing, and as we age, the physiological reserves of several body systems become impaired and dysfunctional [3, 4]. The organism has a balancing and restorative function in response to disorders. However, when homeostasis reaches a certain threshold, weakness occurs and is accompanied by further aging and inflammation [1, 5].

Various chronic lung diseases are associated with ageing [6]. People with lung disease have a higher risk of frailty, developing it decades earlier than healthy people [7]. Frailty is associated with poor prognosis in chronic lung disease, including disease exacerbation, hospitalization, disability and death [8–10]. Cough, sputum, wheezing and breathlessness are common manifestations of lung disease. Even among normal adults, at least half of the population has one respiratory symptom [11, 12]. Treatments targeting respiratory symptoms may be a new target for future pulmonary frailty interventions [7]. Therefore, research addressing the direction and extent of the relationship between respiratory symptoms and frailty is warranted.

The Frailty Index (FI) is one of the key measures of frailty and is composed of the accumulation of multiple health-related deficits [13]. There are few studies on the relationship between respiratory symptoms and frailty. This study aimed to investigate the relationship between respiratory symptoms and FI using two independent but complementary approaches. Data for the observational study were obtained from the National Health and Nutrition Examination Survey (NHANES) 2003–2012. Mendelian randomization (MR) data were derived from genome-wide association studies (GWAS), which are able to simulate the conditions of a randomized controlled trial and help reduce reverse causality [14].

## Methods

### Observational study

#### Data sources

NHANES data collection includes household screenings, interviews, and physical exams [15].

#### Declarations

All the information about this research can be downloaded from the NHANES official website (https://www.cdc.gov/nchs/nhanes/index.htm). The Ethics Committee of the National Centre for Health Statistics approved the NHANES survey study.

#### Respiratory symptoms

Respiratory symptoms include coughing, coughing up sputum, wheezing and dyspnea. Respiratory symptoms are acquired from personal interviews and are defined as follows. Cough was classified as answering yes to the following questions. "Do you usually cough on most days for three consecutive months or more during the year?" to determine. Sputum was classified as answering yes to the following questions. "Do you bring up sputum on most days for 3 consecutive months or more during the year?" to determine. Wheezing and dyspnea were defined as affirmative answers to the following questions. "In the past 12 months, have you had wheezing or whistling in your chest?" and "Have you had shortness of breath either when hurrying on the level or walking up a slight hill?".

#### Frailty index

In the NHANES part of the study, we used the frailty index (FI) developed by Rockwood to assess frailty [13]. FI was calculated using 49 accessible items (Table S1), specifically by dividing the sum of the frailty items by the total measured items (at least 30) [16]. The FI ranges from 0 to 1, with a higher FI indicating greater frailty. Participants with an FI greater than 0.20 were defined as frail and were the primary outcome of this study [17].

#### Inclusion and exclusion criteria

**Fig. 1:**
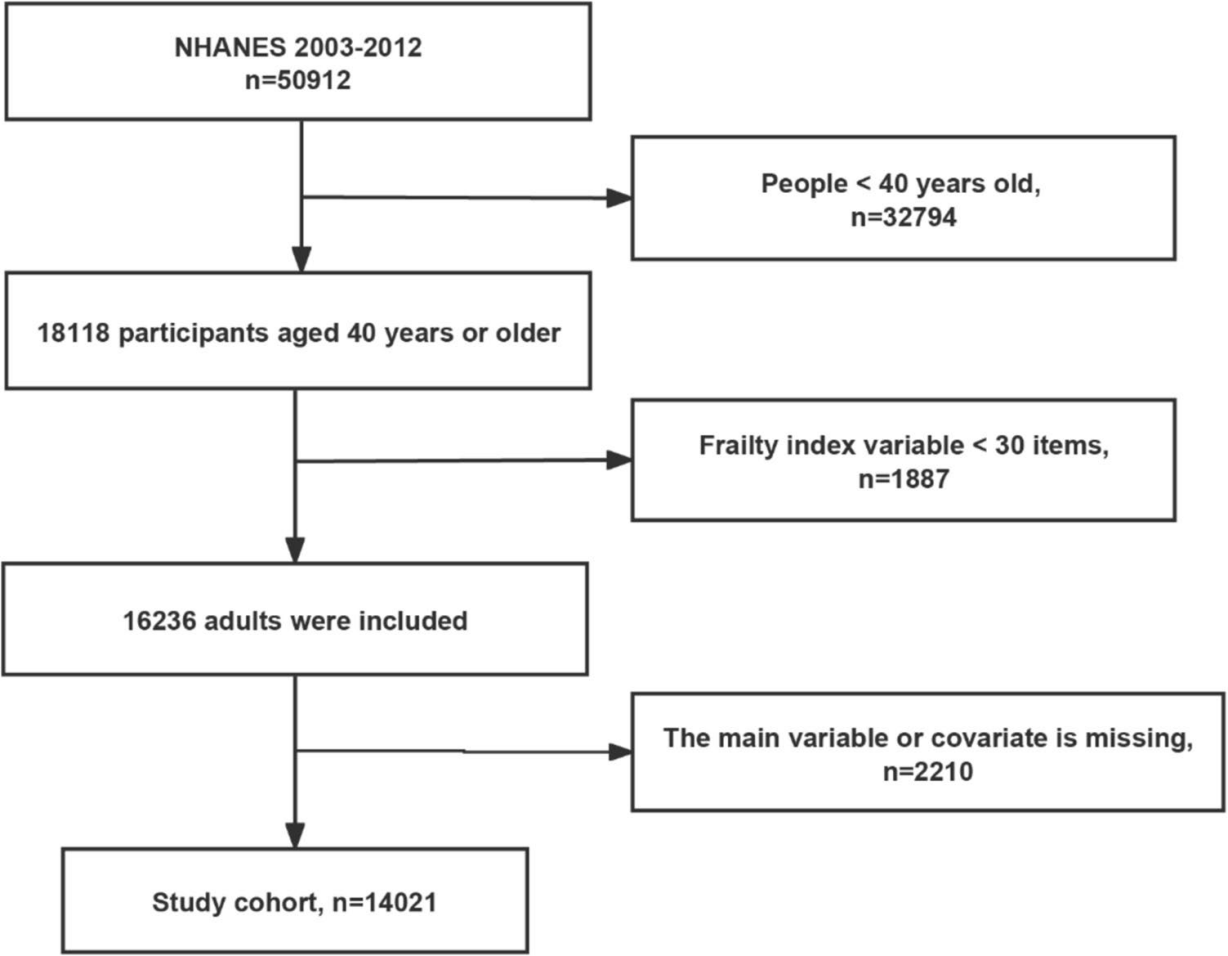
Flow chart of the research

We included participants from NAHNES between 2003 and 2012. The study population consisted of participants undergoing lung health problems and aged ≥ 40 years. We excluded participants whose frailty index variables were less than 30. We also excluded participants who lacked study variables (respiratory symptoms) and who lacked covariates. Detailed inclusion and exclusion results are shown in Fig. 1.

#### Study covariates

We included demographic data to reduce potential bias, including gender, age, body mass index (BMI), race, smoking history, marital status, education, and comorbidities. The race is divided into White, Mexican American, Black and Other races. Smoking history was classified as never (smoking < 100 cigarettes), former (smokes > 100 cigarettes but has quit), and current (still smoking). Comorbidities include diabetes, hypertension, cardiovascular disease, chronic obstructive pulmonary disease (COPD), and asthma, with specific diagnoses described in additional file.

#### Statistical analysis

Continuous data conformed to normal distribution were expressed as mean (standard deviation, SD), and count data were expressed as N (%). Comparisons between variables were made using one-way ANOVA and chi-square tests.

We used multivariate logistic regression to investigate the relationship between respiratory symptoms (cough, sputum, wheeze, dyspnea) and frailty. We adjusted three models to minimize potential bias. The model I adjusted for nothing. Model II adjusted for age, gender and ethnicity. Model III adjusted for age, sex, ethnicity, body mass index, smoking history, education, family income to poverty ratio, hypertension, diabetes, cardiovascular disease, COPD and asthma.

#### Sensitivity analysis

We used 1:1 propensity score matching (PSM) with a caliper set at 0.05 to minimize bias between populations further. We used subgroup analyses to examine the relationship between respiratory symptoms and debility among different subgroups.

#### Mediation analysis

In addition, we performed mediation analyses with the variables of interest being inflammatory markers and age. We included three markers that were available and associated with inflammation, including white blood cells (WBC), neutrophil percentage (NEU, %), and red blood cell distribution width (RDW, %). WBC > 10*10^9^, NEU (%) > 70%, and RDW (%) > 14.5% were defined as elevated levels of inflammation, and age > 60 was defined as higher age. Importantly, RDW and NEU were included among the 49 items for which FI was calculated. we therefore excluded these two indicators, recalculated FI, and then conducted a mediation analysis. In the mediation analysis, 1000 bootstraps were used. The results display values of indirect effects, proportions of mediated effects, and p-values of mediated effects.

The statistical analysis was weighted based on a complex sampling of NHANES (wtmec2yr). This part of the study used R version 4.2.2 and “FreeStatistics”.

### MR study

#### Data sources

In MR analyses, pooled-level data from extensive GWAS were utilized to investigate the potential causal effects of respiratory symptoms (cough, sputum, wheeze, dyspnea) on debility. GWAS for respiratory symptoms were obtained from the IEU database (https://gwas.mrcieu.ac.uk/). Cough was defined as "Cough on most days" (GWAS ID: ukb-b-16858). Sputum was described as "Bringing up phlegm/sputum/mucus on most days" (GWAS ID: ukb-b-12841). Wheezing was defined as "Wheeze or whistling in the chest last year" (GWAS ID: ukb-b-18335). Dyspnea is defined as "Shortness of breath walking on level ground" (GWAS ID: ukb-b-9543). Data for FI were derived from a GWAS meta-analysis, including the UK Biobank (*N* = 164,610) and TwinGene (*N* = 10616) [18]. The FI data can be downloaded from the GWAS catalogue (https://www.ebi.ac.uk/gwas/downloads/summary-statistics; GWAS ID: GCST90020053). The calculation still adds up the items of frailty and divides them by the total number of frailties measured (at least 30) [19].

#### Instrument selection

In this study, we selected a single nucleotide polymorphism (SNP) that was significantly associated with cough, sputum, wheezing, and dyspnea (*P* < 5 × 10–7) as the instrument variant (IV). 5 × 10^–8^ was not chosen for this study because it would have resulted in the study including too few SNPs. Then, to ensure that all IVs satisfy the independence assumption, we use chain imbalance to exclude ineligible IVs (R2 < 0.001 within 10,000 kb clustering distances). The threshold for r2 was established by the 1000 Genomes Project for populations of European origin. In addition, SNP showing palindromic alleles (A/T or G/C) were excluded from the study because of the potential to cause strand disambiguation problems. In addition, we identified SNP with an f-statistic > 10 as strong IV. The specific formula is *F* = R^2^*(N-2)/1-R^2^ and *R*^2^ = 2*β^2^*EAF*(1-EAF) [20]. where R^2^ is the degree of variation explained by the SNP, EAF is the gene frequency of the mutation, β is the coefficient associated with the exposure factor, and N is the total sample size. All the IVs in this study had *F*-values higher than 10, suggesting a strong correlation between them and exposure.

#### Statistical analysis

For MR analysis, we mainly used the inverse variance weighting (IVW) method to analyze the bidirectional causality between respiratory symptoms and FI. We also analyzed the studies using other MR methods, including MR-Egger, weighted median and outliers (MR-PRESSO). Cochrane's Q-values were calculated for each study to assess the presence of heterogeneity. The MR-Egger intercept method was used to determine horizontal multivariate validity. We removed outliers for studies with horizontal multivariate validity < 0.05 and applied the MR-PRESSO method for analysis.

#### Sensitivity analysis

Since the construction of the FI included the wheeze item in the MR analysis, this could lead to bias. Additional frailty measures (frailty phenotype) were included in the sensitivity analysis [21]. We analyzed bidirectional associations between frailty phenotype and respiratory symptoms.

This part of the study applied the MR-PRESSO and the TwoSampleMR package using R v4.2.2.

## Results

### Observational analysis of respiratory symptoms and frailty

#### Baseline characteristics

We included a total of 14,021 participants in our study after excluding missing values and those < 40 years (Fig. 1).

Table 1 shows that 32.3% (4520/14021) of the participants were frail. The participants had a mean age of 61.2 years; 51.6% were white, and 49.5% were male. The frail had a higher Body mass index, a higher proportion of smokers, higher levels of poverty, and higher rates of diabetes, hypertension, cardiovascular disease, and chronic lung disease.

**Table 1:** Population characteristics in frail and non-frail states

Variables	Total (*n* = 14,021)	Non-Frailty (n = 9501)	Frailty (*n* = 4520)	*P*
Age, years, Mean (SD)	61.2 (12.6)	59.8 (12.4)	64.3 (12.7)	< 0.001
Gender, *n* (%)				< 0.001
Female	7075 (50.5)	4516 (47.5)	2559 (56.6)	
Male	6946 (49.5)	4985 (52.5)	1961 (43.4)	
Ethnicity, *n* (%)				< 0.001
Non-Hispanic White	7230 (51.6)	4968 (52.3)	2262 (50)	
Mexican American	2055 (14.7)	1437 (15.1)	618 (13.7)	
Non-Hispanic Black	2931 (20.9)	1804 (19)	1127 (24.9)	
Other Race	1805 (12.9)	1292 (13.6)	513 (11.3)	
Body mass index, *n* (%)				< 0.001
< 25	3663 (26.1)	2686 (28.3)	977 (21.6)	
25–30	5020 (35.8)	3578 (37.7)	1442 (31.9)	
> 30	5338 (38.1)	3237 (34.1)	2101 (46.5)	
Smoke, *n* (%)				< 0.001
Never smoker	6797 (48.5)	4867 (51.2)	1930 (42.7)	
Former smoker	4559 (32.5)	2951 (31.1)	1608 (35.6)	
Current smoker	2665 (19.0)	1683 (17.7)	982 (21.7)	
Marital status, *n* (%)				0.808
Non-Married	1561 (11.1)	1062 (11.2)	499 (11)	
Married	12,460 (88.9)	8439 (88.8)	4021 (89)	
Education, *n* (%)				< 0.001
< High school diploma	2133 (15.2)	1191 (12.5)	942 (20.8)	
≥ high school	11,888 (84.8)	8310 (87.5)	3578 (79.2)	
Family income to poverty ratio, *n* (%)				< 0.001
< 1	2573 (18.4)	1353 (14.2)	1220 (27)	
1–3	6108 (43.6)	3849 (40.5)	2259 (50)	
> 3	5340 (38.1)	4299 (45.2)	1041 (23)	
Comorbidities, *n* (%)				
Hypertension	2545 (18.2)	751 (7.9)	1794 (39.7)	< 0.001
Diabetes	3508 (25.0)	1563 (16.5)	1945 (43)	< 0.001
Cardiovascular disease	8168 (58.3)	4733 (49.8)	3435 (76)	< 0.001
COPD	1314 (9.4)	439 (4.6)	875 (19.4)	< 0.001
Asthma	1762 (12.6)	851 (9)	911 (20.2)	< 0.001
Respiratory systems, *n* (%)				
Cough	1591 (11.3)	727 (7.7)	864 (19.1)	< 0.001
Sputum	1493 (10.6)	678 (7.1)	815 (18)	< 0.001
Wheezing	2117 (15.1)	908 (9.6)	1209 (26.7)	< 0.001
Dyspnea	5078 (36.2)	2395 (25.2)	2683 (59.4)	< 0.001

Table S2 shows that after propensity score matching (PSM), the difference

*COPD* chronic obstructive pulmonary disease

in baseline information between the frail and non-frail participants was insignificant, and the matched data could be used for subsequent analyses. Univariate logistic regression (Table S3) showed that gender, age, race, body mass index, smoking history, education, household income and poverty rate, diabetes, cardiovascular disease, hypertension, chronic obstructive pulmonary disease, and asthma were all associated with frailty.

#### Association between respiratory symptoms and frailty

**Fig. 2:**
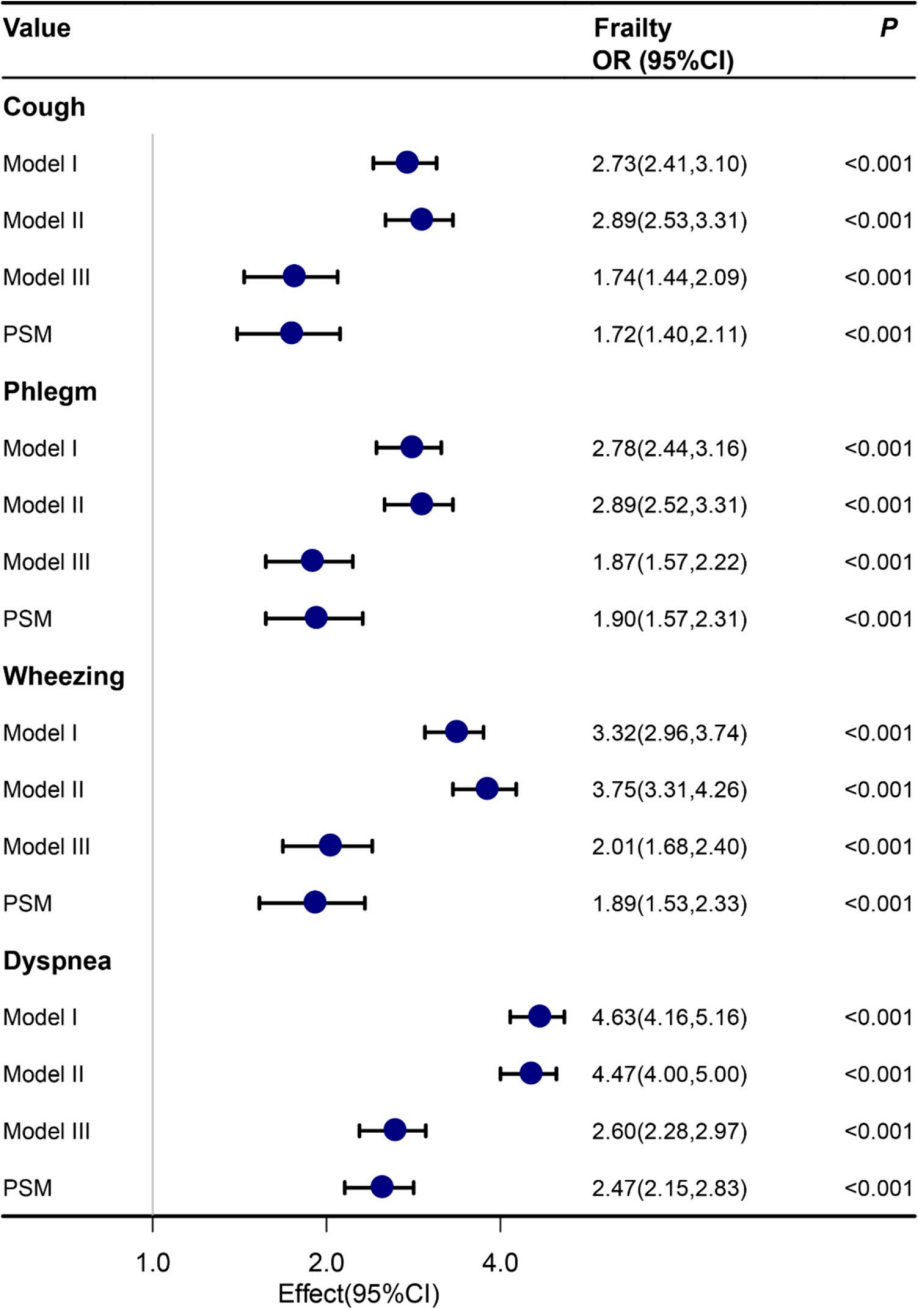
Forest Plot for Association of cough, sputum, wheezing, and dyspnea with the Frailty. Model I adjusted for nothing. Model II adjusted for age, gender and ethnicity. Model III adjusted for sex, age, ethnicity, body mass index, smoking history, education, and family income to poverty ratio, hypertension, diabetes, cardiovascular disease, chronic obstructive pulmonary disease, and asthma (Covariates with *P*<0.05 in univariate analysis)

As shown in Fig. 2, after adjusting for covariates, cough, sputum, wheezing, and dyspnea were associated with elevated FI and increased risk of frailty. In model 3 adjusted for more variables, cough (OR 1.74, 95 CI% 1.44, 2.09, *P* < 0.001), sputum (OR 1.87, 95 CI% 1.57, 2.22,* P* < 0.001), wheezing (OR 2.01, 95 CI% 1.68, 2.40,* P* < 0.001), and dyspnea (OR 2.60, 95 CI% 2.28, 2.97,* P* < 0.001) were significantly associated with an increased risk for frailty. Figure 2 shows that the relationship between respiratory symptoms and frailty was generally consistent before and after PSM.

**Fig. 3:**
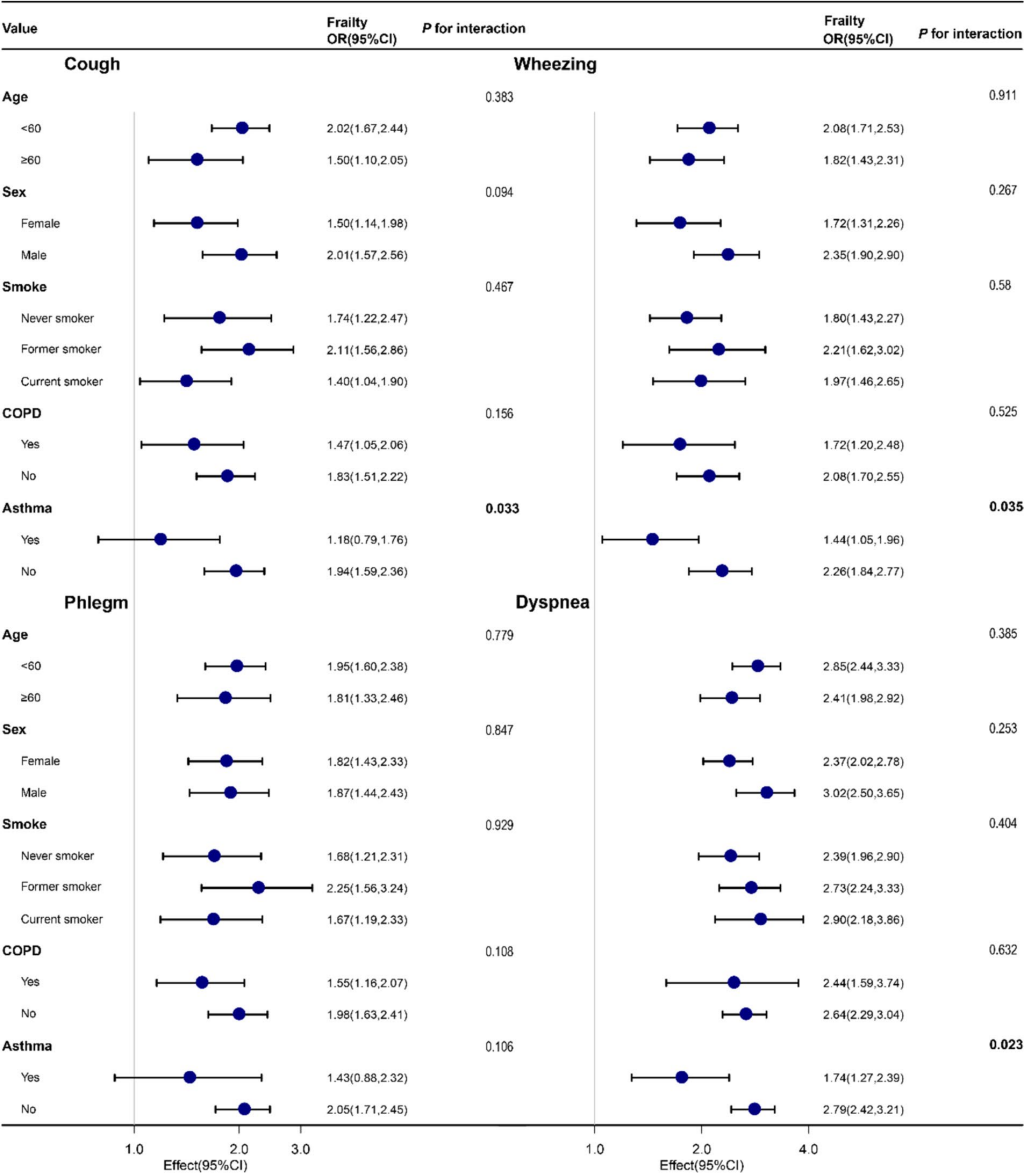
Forest Plot for Subgroup Analyses of the Association Between cough, sputum, wheezing, and dyspnea with the Frailty

The directionality of the effect estimates for all assessment subgroups was consistent with the overall results (Fig. 3).

There was an interaction between cough, wheezing, dyspnea and frailty in asthma (*P* for interaction < 0.05). Non-asthmatic participants with symptoms of cough, wheezing and dyspnea had a higher risk of frailty than asthmatic participants.

Figure S1 shows the mediation analysis of age and inflammatory markers in the relationship between respiratory symptoms and frailty. Increasing levels of inflammation and age mediated the relationship between respiratory symptoms and frailty.

#### MR analysis of respiratory symptoms and frailty

A total of 6 cough-associated, 5 sputum-associated, 72 wheeze-associated, 3 dyspnea-associated, and 41 FI-associated SNPs were screened as genetic instrumental variables (Table S4-S8). As can be seen from the table, the F-values of the SNPs were all greater than 10.

**Fig. 4:**
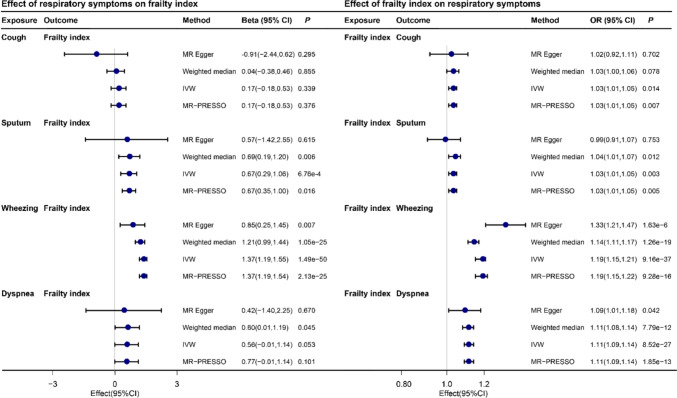
Bidirectional MR analyses for the association between respiratory symptoms and frailty index

Bidirectional causality between respiratory symptoms and frailty index is shown in Fig. 4. The IVW method showed that sputum (Beta 0.69, 95CI% 0.19, 1.20, *P* = 6.76e-04) and wheezing (Beta 1.37, 95CI% 1.19, 1.55, *P* = 1.49e-50) were associated with increased frailty index. There was no significant difference between cough (Beta 0.17, 95CI% -0.18, 1.20, *P* = 0.339), dyspnea (Beta 0.69, 95CI% -0.01, 1.20, *P* = 6.76e-04) and frailty index. On the other hand, an increase in the frailty index was associated with the occurrence of cough (OR 1.03, 95CI% 1.01, 1.05, *P* = 0.014), sputum (OR 1.03, 95CI% 1.01, 1.05, *P* = 0.003), wheezing (OR 1.19, 95CI% 1.15, 1.21, *P* = 9.16e-37), and dyspnea (OR 1.11, 95CI% 1.09, 1.14, *P* = 8.52e-27).

We performed the weighted median and MR-PRESSO methods, and these results remain significant. There is some bias in MR-Egger when the overall effect is in the same direction, which is related to the lower precision of MR-Egger. Although Table S9-S10 shows heterogeneity in Cochran's Q statistic for some associations, this does not affect the final results. In addition, Tables S9-S10 also show that the MR-Egger intercept revealed horizontal pleiotropy in the relationship between frailty index and wheezing symptoms. After screening for exclusion of two SNPs (rs28400568, rss575147125, Table S11) using the MR-PRESSO method, the *P* > 0.05 for horizontal pleiotropy was observed in the relationship between frailty index and wheezing (Table S10). Table S12 shows that the findings were stable after excluding these two SNPs. In addition, Table S13 shows a bidirectional association between the frailty phenotype and respiratory symptoms. Wheezing was associated with elevated FP. Unlike FI, the effect of sputum on FP was not statistically different (*P* > 0.05). And the results of FP on respiratory symptoms were essentially similar to the primary outcome (FI on respiratory symptoms).

## Discussion

To our knowledge, few previous studies have analyzed the relationship between respiratory symptoms and frailty, either in observational studies or in MR. Our study was divided into two parts. The observational study found that respiratory symptoms (cough, sputum, wheezing, and dyspnea) were associated with frailty, which remained stable after adjustment for covariates and PSM. The results of the MR study showed that sputum and wheezing were associated with an elevated frailty index. In the effect of FI on respiratory symptoms, the risk of all respiratory symptoms increased with elevated FI.

Cough, sputum, wheezing and dyspnea are significant complaints of chronic lung diseases and are risk factors for the progression and poor prognosis of many lung diseases [22–24]. In observational studies, frailty has been associated with many chronic lung diseases such as asthma, chronic obstructive pulmonary disease, interstitial lung disease, and lung cancer [10, 25–27]. Our study showed a significant association between frailty and respiratory symptoms. Frailty is a trait closely associated with human ageing, and previous studies have found that respiratory symptoms are associated with accelerated ageing in the population [28]. Respiratory symptoms are related to impaired quality of life even in the general population without COPD and asthma. [29]. Similarly, in the general population, the presence of respiratory symptoms increase the risk of all-cause mortality [30, 31].

In observational studies, we found a remarkable relationship between dyspnea and frailty. Several observational studies have found an association between frailty and impaired lung function [25, 32]. A prospective study showed that frail COPD patients had higher COPD Assessment Test (CAT) and Medical Research Council (MRC) scores than non-frail [33]. An MR result shows an association between impaired lung function and frailty [34]. We found a potential trend between dyspnea and elevated frailty index by MR, but the *p*-value of 0.053 for the IVW method may be related to fewer SNPs reflecting dyspnea. Reverse MR shows that frailty increases the risk of cough and dyspnea. It suggests that improving frailty may positively impact cough and dyspnea.

Both observational studies and MR results showed that sputum and wheezing were associated with frailty index and were bi-directionally correlated. The health consequences of chronic sputum production are enormous, and chronic sputum production is an independent risk factor for accelerated decline in lung function, frequent acute exacerbations and increased mortality in COPD patients [35]. In another study of COPD patients, researchers found that white blood cells in sputum had a higher senescence index than white blood cells in blood [36]. A gradual increase in sputum neutrophils with age was found in two studies of healthy participants and asthmatics [37, 38]. Ageing-related inflammation may be a critical factor in the relationship between sputum and frailty. There are no studies on the relationship between wheezing and frailty. However, wheezing is a significant asthma symptom, and previous MR studies have found a reciprocal causal relationship between frailty and asthma [39]. Another research study found an association between lifelong oral corticosteroid use and frailty in elderly asthmatics [40].

As age increases, the organism's ageing increases the risk of frailty [41]. A meta-study showed people with frail had higher inflammatory parameters, including C-reactive protein, leukocytes, and interleukin-6, than people with non-frail [42]. This result also applies to COPD patients [43]. Combined with the results of mediation analyses (Fig. S1), we suggest that inflammation and ageing play an important role in frailty and respiratory symptoms. Frailty is a dynamic process [44]. Pathophysiologic changes due to chronic inflammation and immune senescence are essential to breaking the frailty balance. Understanding the pathobiology of respiratory symptoms that lead to debilitation is critical, as it allows for targeted early intervention in patients at risk for debilitation. Improving respiratory symptoms (sputum and wheezing) may be essential to reduce the adverse consequences of frailty.

This is the first study to analyze the relationship between respiratory symptoms and frailty indices. The use of mixed models enhances the stability and diversity of the results. On the one hand, the cross-sectional study design influences causal inference, and the use of MR strengthens this interpretation. On the other hand, the data for the MR were mainly derived from European populations, and the use of cross-sectional data from US populations added to the diversity of the study population. Admittedly, the study also has some shortcomings. First, diagnoses of cardiovascular disease, COPD, and asthma are derived from self-reports, which may lead to potential bias. In addition, respiratory symptoms and FI were obtained from patients' self-reports, which may have affected the results. Finally, as the FIs in the UK Biobank and TwinGene (the basis of GWAS) also include several respiratory related items such as asthma, COPD and cough. Therefore, results on MR may be potentially biased. To minimize this concern, we assessed frailty using the frailty phenotype, and the results were similar to FI.

## Conclusions

In summary, through cross-sectional and MR research methods, our study found that respiratory symptoms (sputum, wheezing) increase the risk of frailty. In addition, frailty increases the risk of respiratory symptoms that affect respiratory health. Relevant future studies are needed to explore the potential reasons for the interconnection of respiratory symptoms and frailty.

## Supplementary Information

Below is the link to the electronic supplementary material.

Supplementary file1 (XLSX 53 KB)

Supplementary file2 (DOCX 258 KB)

## Data Availability

Cross-sectional study of data from the NHANES official website (https://www.cdc.gov/nchs/nhanes/index.htm) to download. Mendelian randomization data from GWAS catalogue (https://www.ebi.ac.uk/gwas/downloads/summary-statistics and IEU database (https://gwas.mrcieu.ac.uk/) to download. Data for this article can be obtained by consulting the corresponding author.
