# Supplementary material for: The relationship between respiratory symptoms and frailty: findings from observational and Mendelian randomization analyses
Source: Aging Clin Exp Res. 2024 Dec 30;37(1):17. doi: 10.1007/s40520-024-02905-5 (PMC11685262; doi:10.1007/s40520-024-02905-5)
Supplement: Supplementary file 2 — Supplementary file2 (DOCX 258 KB) [file 40520_2024_2905_MOESM2_ESM.docx]

**Figure S1 Mediation analysis of inflammatory markers and age in the relationship between respiratory symptoms and frailty.**
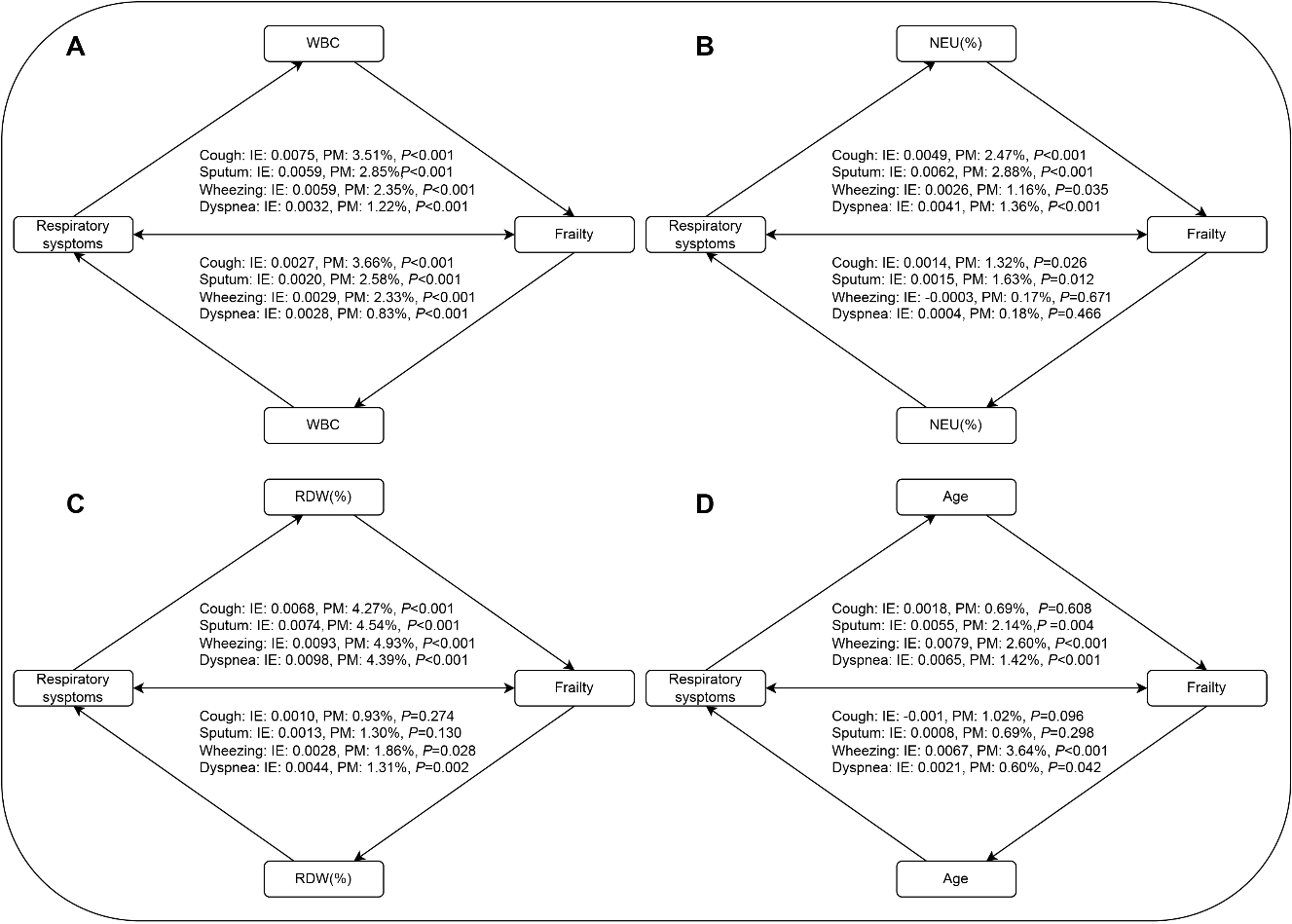


_Figure S1 (A) demonstrates the mediating role of WBC in respiratory symptoms and frailty. Figure S1 (B) demonstrates the mediating role of NEU (%) in respiratory symptoms and frailty. Figure S1 (C) demonstrates the mediating role of RDW (%) in respiratory symptoms and frailty. Figure S1 (D) demonstrates the mediating role of age in respiratory symptoms and frailty._

_Figure S1 (A-C) shows the results after excluding missing data for hematological parameters (13541/14021, 96.58%)._

_Abbreviations: WBC: white blood cell, NEU: neutrophil, RDW: red blood cell distribution width, IE: indirect effect, PM, proportion mediated._
